# E6/E7 Functional Differences among Two Natural Human Papillomavirus 18 Variants in Human Keratinocytes

**DOI:** 10.3390/v13061114

**Published:** 2021-06-10

**Authors:** Emily Montosa Nunes, Valéria Talpe-Nunes, João Simão Sobrinho, Silvaneide Ferreira, Vanesca de Souza Lino, Lara Termini, Gabriela Ávila Fernandes Silva, Enrique Boccardo, Luisa Lina Villa, Laura Sichero

**Affiliations:** 1 Center for Translational Research in Oncology, Instituto do Cancer do Estado de São Paulo (ICESP), Hospital das Clinicas da Faculdade de Medicina da Universidade de Sao Paulo (HCFMUSP), São Paulo 01246-000, Brazil; emontosa.biotec@gmail.com (E.M.N.); valeria.talpe.nunes@gmail.com (V.T.-N.); simao.sob@gmail.com (J.S.S.); silvaneide.ferreira@hc.fm.usp.br (S.F.); terminilara@gmail.com (L.T.); gabi-avila@hotmail.com (G.Á.F.S.); l.villa@hc.fm.usp.br (L.L.V.); 2 Department of Microbiology, Instituto de Ciências Biomédicas, Universidade de Sao Paulo, São Paulo 05508-900, Brazil; vanesca_lino@hotmail.com (V.d.S.L.); eboccardo@usp.br (E.B.); 3 Department of Radiology and Oncology, Faculdade de Medicina, Universidade de Sao Paulo, São Paulo 01246-000, Brazil

**Keywords:** HPV-18, oncogenic potential, molecular variant, cell transformation, immortalization

## Abstract

It is suggested that HPV-18 variants from the A lineage have higher oncogenic potential compared to B variants. Some studies show uneven distribution of HPV-18 variants in cervical adenocarcinomas and squamous cell carcinomas. Regarding HPV-18 variants’ functions, the few studies reported focus on E6, and none were performed using natural host cells. Here, we immortalized primary human keratinocytes (PHKs) with E6/E7 of HPV-18 A1 and B1 sublineages and functionally characterized these cells. PHK18A1 reached immortalization significantly faster than PHK18B1 and formed a higher number of colonies in monolayer and 3D cultures. Moreover, PHK18A1 showed greater invasion ability and higher resistance to apoptosis induced by actinomycin-D. Nevertheless, no differences were observed regarding morphology, proliferation after immortalization, migration, or epithelial development in raft cultures. Noteworthy, our study highlights qualitative differences among HPV-18 A1 and B1 immortalized PHKs: in contrast to PHK18A1, which formed more compact colonies and spheroids of firmly grouped cells and tended to invade and migrate as clustered cells, morphologically, PHK18B1 colonies and spheroids were looser, and migration and invasion of single cells were observed. Although these observations may be relevant for the association of these variants with cervical cancer of different histological subtypes, further studies are warranted to elucidate the mechanisms behind these findings.

## 1. Introduction

Human papillomavirus (HPV) responds for virtually all cases of cervical cancer (CC). Worldwide, cervical squamous cell carcinomas (SCCs) are primarily associated with HPV-16 infection (60%), while HPV-16 and -18 are similarly prevalent in adenocarcinomas (ADCs) [1]. Based on a fragment of the long control region (LCR), HPV-18 genetic variants were initially classified into three branches of phylogenetic and geographical relatedness: African (Af), Amerindian (As + AI or American Indian or East Asian), and European (E) [2]. More recently, the nomenclature of HPV-18 variants was revised, and these are now categorized into variant lineages (1–2% sequence difference; designated by letters) and sublineages (0.5–1% sequence difference; designated by numbers) based on whole-genome sequencing. Thus, now while A1/A2 and A3/A4 HPV-18 sublineages correspond to previously nominated As + AI and E variants, respectively, the HPV-18 B lineage comprises previously nominated Af variants [3,4].

HPV-16 Asian American (AA) variants are clearly associated with a higher risk of cervical neoplasia and cancer development [5,6] and a higher oncogenic potential in vitro [7,8]. However, for HPV-18 variants, data are still conflicting. Studies conducted in Mexico [9], Brazil [8], Portugal [10], and the USA [11] indicated that HPV-18 A lineage variants are epidemiologically associated with increased oncogenic potential. Instead, reports from Spain [12], Costa Rica [13], the USA [14], and from a multinational collection of samples [3] did not show similar trends. Noteworthy, in some studies, HPV-18 B lineage variants were exclusively detected in SCC, whereas A lineage variants were more prevalent in ADC [9,15,16].

High-risk (HR) HPV E6/E7 proteins cooperate to immortalize primary human keratinocytes (PHKs) and to inhibit their differentiation induced by serum and calcium [17]. E6 and E7 interact with several cellular proteins, including *p*53 and pRB, respectively, leading to dysregulation of apoptosis, proliferation, and senescence [18]. Indeed, organotypic cultures established from HPV-18 positive PHKs present histological alterations similar to human intraepithelial cervical lesions [19,20]. We and others showed that HPV-18 A1 sublineage variants have higher transcriptional activity than B1 lineage isolates [21,22]. Furthermore, few studies indicate that functionally HPV-18 A1 sublineage variants have a higher oncogenic potential [23,24,25]. However, no studies comparing HPV variants to date were performed in the background of HPV-18 natural host cells (primary human keratinocytes, PHK). Moreover, all studies focused solely on E6. Here, we characterized the biological properties of PHKs immortalized by E6/E7 of HPV-18 A1 and B1 sublineage variants. We observed functional and phenotypical differences between PHKs transduced with HPV-18 variants that may contribute to explain their role in CC and the association with different histological subtypes.

## 2. Materials and Methods

### 2.1. Cells and Plasmids

HeLa cells (lot 59681574, ATCC CCL-2, American Type Culture Collection, Manassas, VA, USA), (obtained from ATCC in 2015 but used here at *p*3) were grown in DMEM (Invitrogen, Carlsbad, CA, USA), 10% FBS, as previously reported [26]. Two distinct batches of pooled primary human foreskin keratinocytes (PHK) (Lonza Group Ltd., Basel, Switzerland, #00192906, batches #355184 [pool 1] and 357479 [pool 2]) also commercially obtained in 2016 but used here at *p*0 were maintained in keratinocyte serum-free medium (KSFM, Invitrogen) supplemented with 5 ng/mL EGF (Invitrogen) and 50 mg/mL BPE (Invitrogen). Complete HPV-18 *E6*/*E7* genes of A1 and B1 lineage variants were amplified from previously characterized cervical swabs [5], cloned within the pLNSX retroviral vector (GenBank M28246.1) and sequenced using the BigDye Terminator v3.1 Cycle Sequencing Kit and the ABI 3130XL Genetic Analyzer (both Applied Biosystems, Foster City, CA, USA).

### 2.2. PHK Growth Kinetics

PHKs were transduced as previously described [26], using equivalent amounts of retroviral particles (MOI = 10). These were further selected with 300 µg/mL G418 for two weeks and then pooled and subcultured weekly (1:6). Once PHKs transducing empty pLNSX ceased proliferation at *p*5, nontransduced PHKs were used as controls throughout experiments. Growth kinetics was evaluated by consecutive plating 1.25 × 10^5^ cells in 25 cm^2^ bottles, subculturing at 80% confluence, and counting using the Countess Cell Counter (Invitrogen). At *p*30 cells were considered immortalized. Doubling time was calculated at http://www.doubling-time.com/compute.php (accessed on 3 April 2020).

### 2.3. Cell Proliferation

Cells were seeded at 5 × 10^3^ cells in 12-well plates and counted after two, four, and six days. Cells were then fixed, kept at −20 °C for one hour, washed with 1 × PBS, and incubated with anti-Ki67 (ab92742, abcam, Cambridge, UK) at room temperature for one hour, followed by incubation with Alexa Fluor 633-secondary antibody (A-21070, Thermo Scientific, Waltham, MA, USA). Flow cytometry was conducted using the Attune Acoustic Focusing Flow Cytometer (Thermo Scientific, Waltham) and data analyzed using FlowJo X 10.0.7r2 (Tree Star, Ashland, OR, USA).

### 2.4. qRT-PCR

Total RNA was extracted using Trizol (Invitrogen) and quantified using NanoDrop One (Thermo Scientific, Waltham, MA, USA). qRT-PCRs using GoTaq 1-Step RT-qPCR System (Promega, Madison, WI, USA) and the 7500 Real-Time PCR System (Applied Biosystems) were performed to access the expression of *E6* + E6*I (nts 111–180), *E6* (506–578), and *E7* (750–852) (Supplementary Table S1). Mitochondrial ribosomal protein S18 (192–339) mRNA levels were used as control and relative expression calculated, as described [27].

### 2.5. Western Blotting

Cell lysates were obtained using RIPA (20 mM Tris-HCl pH = 7.5, 150 mM NaCl, 0.5% sodium deoxycholate, 0.1% SDS, 1% NP40) containing protease and phosphatase inhibitors (Roche, Basel, Switzerland). A total of 60–120 μg protein were fractionated by SDS–PAGE and transferred to PVDF membranes (GE Healthcare Life Sciences, Buckinghamshire, UK). These were blocked in 5% nonfat dry milk in TBS-T (20 mM Tris-HCl pH = 7, 5, 150 nM NaCl, 0,1% Tween20) for one hour and immunoblotted. Blots were developed using ECL Detection Reagent (GE Healthcare Life Sciences), images acquired on ImageQuant LAS 4000 (GE Healthcare Life Sciences) and analyzed using Image J 1.52p (National Institute of Health, Bethesda, MD, USA). Primary antibodies were used against HPV-18 E6 (AVC #1006, Arbor Vita Corporation, Fremont, CA, USA), p53 (sc-126, Santa Cruz, Dallas, TX, USA), pRb (ab24, abcam), p16INK4a (ab16123, abcam, Cambridge, UK), *p*21 (ab7960, abcam), PCNA (ab29, abcam, Cambridge, UK) and α-tubulin (T9026, Sigma-Aldrich, St. Louis, MO, USA).

### 2.6. DNA-Induced Damage and Apoptosis

Cells were grown until 80% confluence in 100 mm plates and incubated with 0.5 nM actinomycin-D (AD) (Sigma-Aldrich) for 24 h. Cells were fixed, washed, resuspended in 0,1% TritonX100, RNase 200 µg/mL (Invitrogen), and propidium iodide 20 µg/mL (Thermo Scientific), and analyzed by flow cytometry. Cells were also seeded at 5 × 10^3^ in 12-well plates, and after six days, 0.5 nM AD was added for 24 h. Caspases 3 and 7 levels were accessed using CellEvent Green ReadyProbes Reagent (Invitrogen).

### 2.7. EMT (Epithelial–Mesenchymal Transition) Analysis

Cells were seeded at 2 × 10^4^ in 6-wells low-attachment plates (Corning Inc., Corning, NY, USA). After 14 days, spheroids were collected by centrifugation, washed, and incubated with Versene buffer (EDTA 1mM, NaCl 0.17M, KCl 3mM, Na_2_HPO_4_ 1.6mM pH = 7.2). Cells were incubated with PE-anti-E-cadherin (562870, BD Biosciences, San Jose, CA, USA) and Alexa Fluor 488-anti-vimentin (562338, BD Biosciences), or PE-Mouse IgG1-Isotype Control (554680, BD Biosciences,), and Alexa Fluor 488-Mouse IgG1 Isotype Control (557721, BD Biosciences) for 40 min in ice, followed by flow cytometry.

### 2.8. Clonogenic Assay

Cells were seeded at 500 cells in six-well plates. After 10 days, cells were stained with crystal violet (Sigma-Aldrich). Throughout this study, images were recorded macroscopically using the UVD GelDoc-It 310 Imaging System (Thermo Scientific) and microscopically using Primo Vert microscope (Zeiss, Jena, Germany) or EVOS FL Cell Imaging System (Life Technologies, Carlsbad, CA, USA), and evaluated using Image J 1.52p (National Institute of Health, Bethesda, MD, USA).

### 2.9. Induction of Differentiation

Cells were seeded at 1 × 10^3^ in six-well plates and, after seven days, maintained or not in a differentiation-inducing medium (DMEM, 10% FBS, 1 µg/mL hydrocortisone) for one week. Cells were incubated with 0.5 mg/mL MTT (Sigma-Aldrich) for three hours, diluted in DMSO, and absorbance measurements at 540 nm were performed.

### 2.10. Spheroid Formation Assay

Cells were seeded at 2 × 10^4^ in six-wells low-attachment plates (Corning Inc., Corning, New York, NY, USA) and maintained in culture for 14 days. Cells were also seeded at 5 × 10^3^ in 0.5% soft agar (Invitrogen) in 24-well plates. After 15 days, colonies were stained with 1 mg/mL MTT (Sigma-Aldrich). Finally, cells were grown until 80% confluence and incubated with Nanoshuttle-PL magnetic nanoparticles (Greiner Bio-One GmbH, Frickenhausen, Germany) for 16 h. Cells were then seeded at 1 × 10^4^ cells in a 96-well low-attachment plate (Greiner Bio-One GmbH, Frickenhausen, Germany), and the spheroid drive magnetic plate was placed underneath for 24 h to induce spheroid formation, which was further monitored for 15 days.

### 2.11. Cell Migration and Invasion

Cells were plated at 5 × 10^5^ in 12-well plates. After 24 h, cells were incubated with 10 µM mitomycin C (Sigma-Aldrich) and, scrapes were performed throughout wells. To ensure cells stopped proliferating due to mitomycin C, cells were fixed, incubated with propidium iodide, and analyzed by flow cytometry. The invasion was evaluated using the QCM High sensitivity non-cross-linked collagen invasion assay (Millipore, Darmstadt, Germany) following the manufacturer’s guidelines. Cells were seeded at 2.5 × 10^5^ within upper chambers in nonsupplemented KSFM and as chemoattractant KSFM 15% FBS was added to lower chambers. After 72 h, inserts were stained and immersed in an extraction medium, and absorbance measurement at 540 nm was performed.

### 2.12. Raft Cultures and Immunohistochemistry

Immortalized PHK18A1 and PHK18B1, and parental PHKs at *p*0 were submitted to epithelial raft cultures, as described [28]. Briefly, parental PHK, PHK18A1, and PHK18B1 were seeded on top dermal equivalents (2 × 10^5^ cells/equivalent) composed of rat tail type 1 collagen (Corning Inc., Corning, NY, USA) and 3T3-J2 fibroblasts. After 24 h, the rafts were transferred to the medium–air interface and maintained for 9 days to allow cell growth and tissue stratification. We performed two independent raft experiments with at least six replicates. Epitheliums were fixed in formaldehyde 2%, paraffin-embedded, and tissue sections obtained for histological analysis or immunohistochemistry (IHQ). Sections were probed for cytokeratin 10 (CK10) (ab76318, abcam), p16 (sc-56330, Santa Cruz, Dallas, TX, USA), and PCNA (ab29, abcam) using the Novolink Max Polymer Detection System (Leica Biosystems, Wetzlar, Germany). CC and normal epithelia were used as positive control samples for p16 and CK10 immunostaining, respectively.

### 2.13. Statistical Analysis

All analyses were conducted using the Statistix 8 program for Windows (Analytical Software, Tallahassee, FL, USA). The *T*-test was used to compare results obtained for nontransduced PHKs to those of PHK18A1 and PHK18B1. Significance levels were set at 0.05.

## 3. Results

### 3.1. E6/E7 of HPV-18 Variants Differ in Immortalization Ability

**Figure 1:**
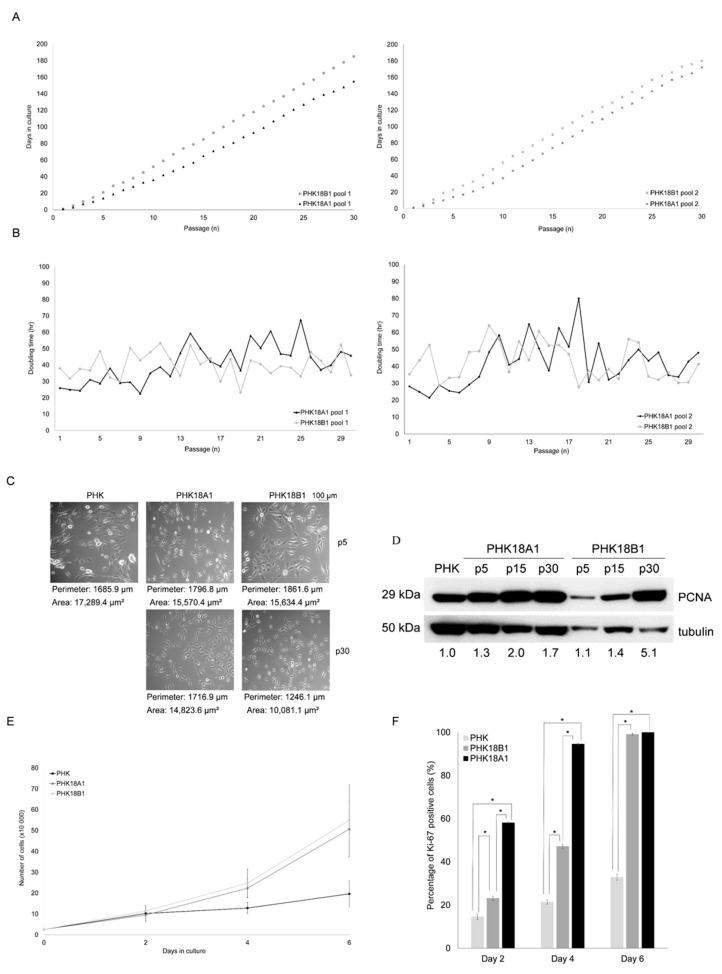
Proliferation kinetics of PHKs transducing *E6/E7* of HPV-18 A1 (PHK18A1) or B1 (PHK18B1) sublineage variants: (**A**) number of passages per day in culture using two different batches of PHK pools. Each point represents a passage in culture. The same number of cells was plated at each passage. *T*-test was used to compare the two different PHK batches transducing the same HPV-18 variant, or to compare the same batch of PHKs transducing the different variants; (**B**) doubling time in hours of PHK18A1 and PHK18B1 along passages in culture; (**C**) morphology of parental PHK#1 (*p*5), PHK18A1, and PHK18B1 in low passage (*p*5) and after immortalization (*p*30) in 10× magnification; (**D**) PCNA levels. Overall, 60 µg of protein extracts were fractionated in SDS-PAGE and PCNA levels were accessed using Western blotting. Tubulin levels were evaluated for loading control. A representative of three independent assays is presented; (**E**) proliferation rates. Cells counting was performed every other day for six days. Means and the standard error of three experiments carried out independently in triplicate are presented; (**F**) percentage of Ki-67 positive cells over six days in culture. Cells from (**E**) were fixed, incubated with anti-Ki-67, and analyzed by flow cytometry. * *p* = 0.00.

Comparing to E6/E7 of HPV-18 A1 sublineage variant, only one nonconservative change was observed in each gene (*E6*: N129K and *E7*: H2Y) of the B1 sublineage variant (Supplementary Table S2). Two distinct PHK pools were transduced with pLNSX E6/E7 of HPV-18 A1 or B1 sublineage variants (named PHK18A1 and PHK18B1, respectively, hereinafter). At *p*5, the DNA of transduced PHKs was isolated, and adequate *E6* and *E7* sequences were confirmed by sequencing. Cells were next subcultured until *p*30, when they were considered immortalized. Parental PHKs and empty vector transduced PHKs ceased proliferation at passages nine and five, respectively. PHK18A1 and PHK18B1 pools reached passage 30 on average after 163 and 182 days in culture, respectively (Figure 1A). For both batches of PHK pools used, PHK18A1 reached *p*30 significantly faster than PHK18B1 (*p* = 0.000). No significant differences were observed between the two pools of PHKs transduced with the same HPV-18 variant (batch 1 versus batch 2; PHK18A1: *p* = 0.53; PHK18B1: *p* = 0.29). Although differences between variants regarding doubling time were more evident before *p*10, an evident crisis during immortalization was not observed either for PHK18A1 or PHK18B1, independently of the batch of cells used (Figure 1B). No differences were observed between PHK18A1 and PHK18B1 in morphology, cell area (PHK18A1 versus PHK18B1: *p*5: *p* = 0.98; *p*30: *p* = 0.5), or perimeter (PHK18A1 versus PHK18B1: *p*5: *p* = 0.69; *p*30: *p* = 0.55). Nevertheless, both transduced cells were significantly smaller than parental cells in area (PHK versus PHK18A1: *p*5: *p* = 0.01, *p*30: *p* = 0.000; PHK versus PHK18B1: *p*5: *p* = 0.01, *p*30: *p* = 0.01), and perimeter (PHK versus PHK18A1: p5: *p* = 0.03, *p*30: *p* = 0.02; PHK versus PHK18B1: *p*5: *p* = 0.02, *p*30: *p* = 0.00) (Figure 1C).

### 3.2. Immortalized PHK18A1 and PHK18B1 Show No Difference in Proliferation

Since no differences were observed between the two batches of transduced PHKs with the same HPV-18 lineage variant concerning the time required to reach *p*30, doubling time, and cell morphology, we decided to follow our analysis using solely transduced PHK pool 1. The proliferation rate of immortalized PHKs (*p*30) was assessed using three distinct approaches as follows. For both immortalized PHKs, we observed an increase in PCNA levels along passages, which was, however, more evident for PHK18B1 (Figure 1D). Furthermore, despite we observed that both immortalized PHKs proliferated significantly faster than parental cells (PHK18A1 versus PHKs: *p* = 0.00; PHK18B1 versus PHKs: *p* = 0.01), proliferation rates were not significantly different between variants (*p* = 0.38) (Figure 1E). Finally, we observed that in line with Figure 1E, Ki-67 protein levels also increased in cells over time in culture, but no significant difference was observed between variants after 6 days in culture (*p* = 0.19) (Figure 1F). Taken together, our data show that while PHK18A1 reached immortalization at an earlier time point, compared to PHK18B1, after immortalization, these cells proliferated at a similar ratio.

### 3.3. PHK18A1 and PHK18B1 Exhibit Similar E6*I, E6, E7, p53, p16, pRb, and p21 levels

**Figure 2:**
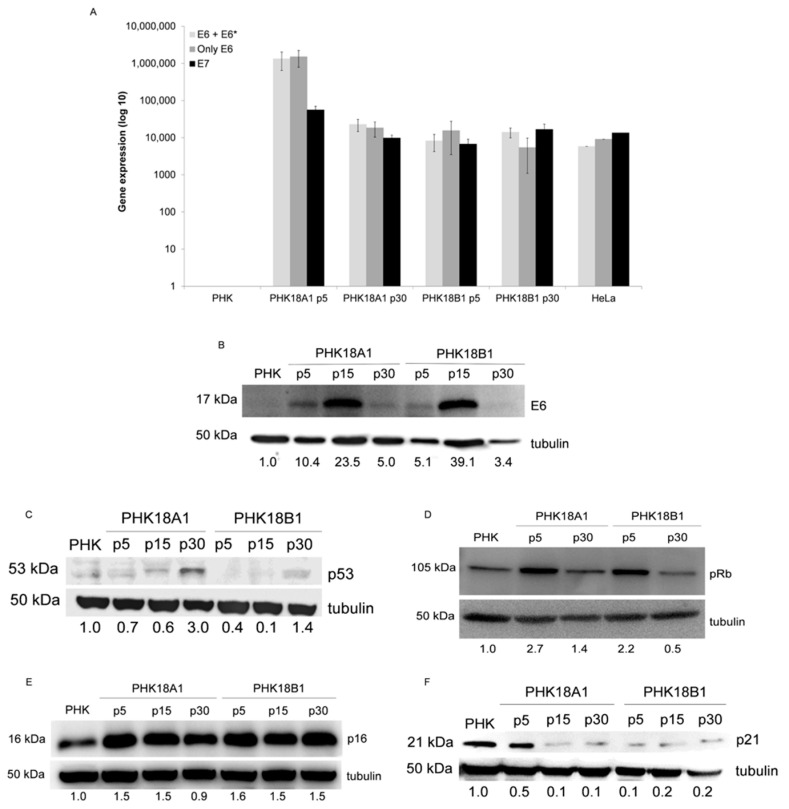
E6, E6*I, E7, p53, p16, pRb, and p21 levels in PHK18A1 and PHK18B1: (**A**) qRT-PCR to quantify E6 + E6*I, E6 alone, and E7 expression. Mitochondrial ribosomal RNA S18 gene expression was used for normalization. Means and standard errors of three independent assays performed in triplicate is presented; (**B**) E6 protein levels. A total of 120 μg of protein extracts were fractionated in SDS–PAGE and the levels of E6 were analyzed by Western blotting; (**C**) p53, (**D**) pRb, (**E**) p16, and (**F**) p21 protein levels. A total of 80 μg of protein extracts were fractionated in SDS–PAGE and protein levels were analyzed by Western blotting. For (**B**–**F**), tubulin levels were used as loading control. Representative experiments of two assays are presented.

The reason why the E6*I transcript is produced in HR-HPVs infected cells is not completely understood. However, it has been shown that the alternative processing that results in increased E6*I levels favors E7 expression [29]. Among HR viral types, HPV-18 infected cells exhibited the highest levels of E6*I [30]. Here, we observed similar levels of E6 + E6*I, E6, and E7 transcripts in cells transduced with HPV-18 E6/E7, regardless of HPV-18 sublineage variant or culture passage (Figure 2A).

Since the correlation between the amount of a transcript and the corresponding protein is not always direct, E6 protein levels were further evaluated. We observed that PHK18A1 and PHK18B1 similarly exhibited lower levels of E6 in passages 5 and 30 compared to those of passage 15 (Figure 2B). Unfortunately, under our conditions, we were unable to detect the E6*I protein in any of the samples. p53 levels inversely correlated with those of E6 in HPV-18 transduced PHKs (Figure 2C).

HR-HPVs E7 represses the tumor suppressor pRb leading to *p*16 overexpression in cervical tumors [31]. Here, HPV-18 E7 protein levels were indirectly measured by accessing the cellular targets pRb, p16, and p21. We observed that pRb levels were reduced in immortalized PHK18A1 and PHK18B1 (*p*30), in comparison with early passage cells (*p*5) (Figure 2D). p16 and p21 levels were also comparable among variants (Figure 2E,F).

### 3.4. PHK18A1 and PHK18B1 Similarly Overcome Actinomycin-D (AD)-Induced Growth Arrest

**Figure 3:**
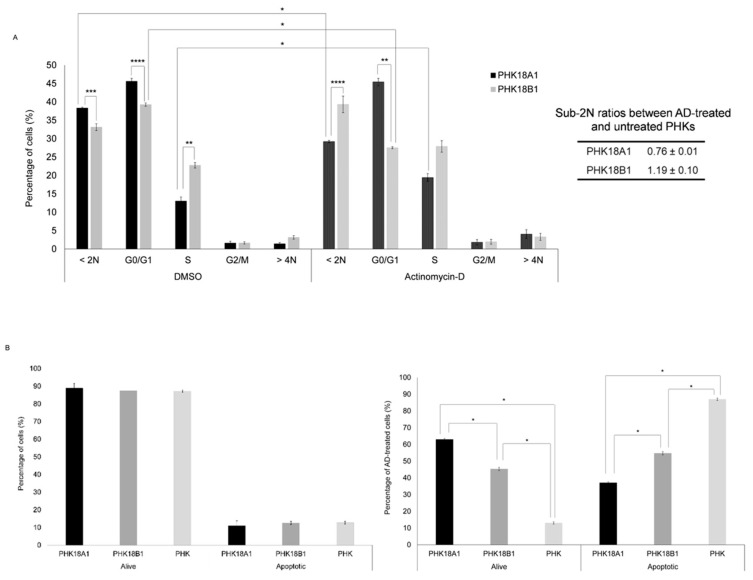
Response of immortalized PHK18A1 and PHK18B1 to actinomycin-D (AD) treatment: (**A**) Cell cycle arrest response to AD treatment. Cells were incubated with AD (0.5 nM) or DMSO for 24 h before incubation with propidium iodide 20 µg/mL. The distribution of cells in each phase of the cell cycle was accessed by flow cytometry. Average of three independent experiments conducted in triplicate; (**B**) apoptosis response to AD treatment. Cells were treated or not with AD (0.5 nM) for 24 h and after six days the activities of caspases 3 and 7 were evaluated by flow cytometry. The *T*-test was used for statistical analysis. * *p* = 0.00, ** *p* = 0.01, *** *p* = 0.02, **** *p* = 0.04.

We observed that AD treatment induced a significant decrease in the percentage of PHK18B1 in G0/G1 and an increase in the rate of PHK18A1 cells in the S phase (Figure 3A). Interestingly, the sub-2N ratio between AD treated and untreated cells was significantly higher for PHK18B1, compared to PHK18A1 (*p* = 0.04), indicating that PHK18B1 were more prone to suffer from AD-induced cell death. Thus, we accessed the levels of caspases 3 and 7 in these cells. Using this approach, we observed no difference in the rate of apoptotic cells between untreated PHK18A1 and PHK18B1 (*p* = 0.35) (Figure 3B). However, corroborating cell cycle analysis, following AD treatment the ratio of apoptotic cells was significantly higher for PHK18B1 (54.7%), compared to PHK18A1 (37.1%) (*p* = 0.00). Taken together, our data indicate that both PHK18A1 and PHK18B1 were capable of overcoming growth arrest induced by AD treatment, although PHK18B1 were significantly more prone to apoptosis following treatment.

### 3.5. PHK18A1 and PHK18B1 Differ in Colony Formation Ability in Monolayer Cultures

**Figure 4:**
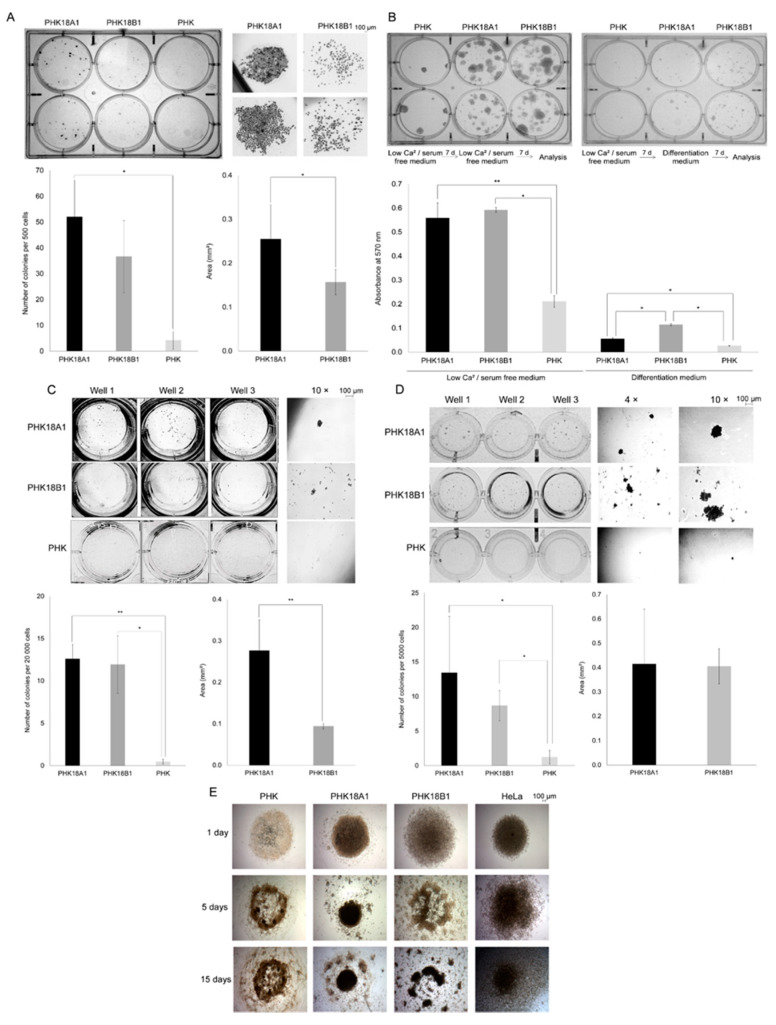
Colony and spheroid formation by immortalized PHK18A1 and PHK18B1 in monolayer and 3D cultures: (**A**) colony formation efficiency after low-density plating. Seven days following plating, cells were fixed and stained with crystal violet. Representative plates from three independent experiments are shown, in addition to an example of colonies formed in 4× magnification. Histograms show the average number and area of colonies formed. * *p* = 0.00; (**B**) efficiency of colonies formation resistant to differentiation induced by serum and calcium. Seven days following plating, culture medium was either maintained (control) or replaced for a differentiation-inducing medium. After one week, colonies were stained with MTT. Cells were then diluted in DMSO and absorbance measured at 570 nm. Images illustrate a representative out of three independent experiments. * *p* = 0.00, ** *p* = 0.01; (**C**) efficiency of spheroid formation in low-attachment plates. Fourteen days following plating, cells were imaged. Representative wells obtained from three independent experiments conducted in triplicate, in addition to 10× magnification images are shown Histograms show the average number and area of spheroids formed. * *p* = 0.00, ** *p* = 0.01; (**D**) efficiency of colony formation in semisolid medium. Cells were grown in 0.5% soft agar in triplicates. After 15 days, cells were stained with MTT. Images illustrate representative wells from three independent experiments, in addition to images of the colonies under 4× and 10× magnification. Histograms show the average number and area of colonies formed. * *p* = 0.00; (**E**) efficiency of spheroid formation through magnetic bioprinting of cells. Cells were incubated with Nanoshuttle-PL solution and further seeded in low-attachment plates on top of a magnetitic spheroid driver for 24 h. Spheroid’s growth was monitored for 15 days. Solely one experiment was conducted with an average of 20 replicates for each cell line evaluated. The *T*-test was used in the statistical analysis.

The ability of immortalized PHKs in forming colonies in monolayer cell culture was accessed using two approaches: clonogenic assay and capacity to resist differentiation induced by serum and calcium. Although not statistically significant, PHK18A1 induced the growth of a larger number of colonies (average of 52 colonies) when compared to PHK18B1 (average of 36.7 colonies) (*p* = 0.13), (Figure 4A). It is noteworthy that colonies formed by PHK18B1 were less cohesive and composed of a reduced number of cells, in comparison to those of PHK18A1, which originated more dense colonies of firmly grouped cells. Indeed, the average area of PHK18A1 (0.25 mm^2^) colonies was significantly higher than that of PHK18B1 (0.16 mm^2^) (*p* = 0.00).

Terminal differentiation was induced by maintaining cells in DMEM supplemented with 10% FBS and 1 µg/mL of hydrocortisone. We observed that under these conditions, PHK18B1 showed a greater ability to abrogate differentiation in this condition, compared to PHK18A1 (*p* = 0.00) (Figure 4B).

### 3.6. PHK18A1 and PHK18B1 Differ in the Ability to form Colonies in 3D Cultures

Initially, cells were plated in low-attachment plates and spheroids formation and growth were monitored. After 14 days, we observed no differences in the number of spheroids formed by PHK18A1 (average of 12.6 spheroids), compared to PHK18B1 (average of 11.9 spheroids) (*p* = 0.70) (Figure 4C). However, similar to what we observed in the monolayer (Figure 4A), spheroids derived from PHK18B1 were also much less cohesive when compared to those of PHK18A1. The mean area of PHK18A1 derived spheroids (0.28 mm^2^) was significantly higher than those resulting from PHK18B1 (0.09 mm^2^) (*p* = 0.01).

We further seeded cells in low melting soft agar and after 15 days colonies were stained. Consistent with the clonogenic assay (Figure 4A), PHK18A1 were more prone to form colonies compared to PHK18B1 (mean number of colonies PHK18A1 versus PHK18B1: 13.4 versus 8.7; *p* = 0.10) (Figure 4D). Nevertheless, in this assay, the average colonies area was similar among variants (*p* = 0.67).

Finally, the ability of cells in forming spheroids was evaluated using a recently developed magnetic cell bioprinting technology (n3D Biosciences). Using this assay, a single spheroid per well is formed. Spheroids formed by PHK18B1 had a porous aspect, similar to parental PHKs but very different from those derived from PHK18A1 or HeLa (Figure 4E). Even though these results originate from a single experiment in which an average of 20 replicates was performed for each cell line studied, they corroborate and expand data obtained in monolayer and the other 3D culture assays (Figure 4A–D).

### 3.7. PHK18A1 and PHK18B1 Differ in Invasion but Not in Migration Ability

**Figure 5:**
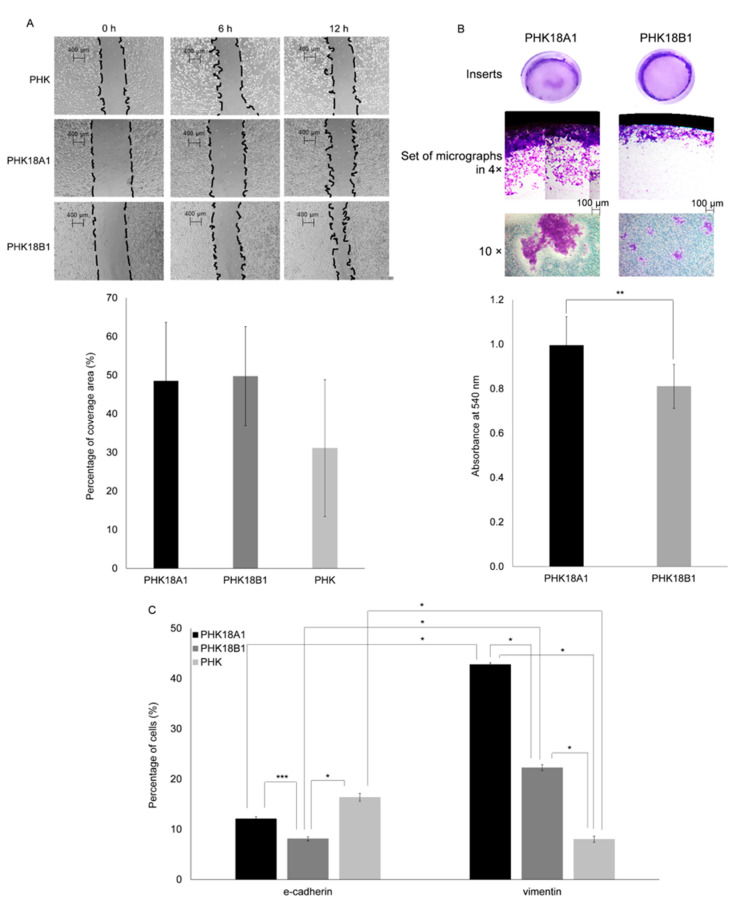
Migration and invasion potential, and EMT phenotype of immortalized PHK18A1 and PHK18B1: (**A**) wound healing assays. Cells were seeded and a wound was made throughout the center of the well. Images illustrate representative wells from three independent experiments carried out in triplicate. Histograms show the average area covered 12 h after wounding; (**B**) invasion assay. Cells were plated in collagen-covered inserts in nonsupplemented KSFM and invasion was stimulated by adding KSFM 15% FBS outside the inserts. Cellular invasion was monitored for 72 h. Images are representative of three independent experiments conducted in triplicate. Histograms show the average absorbance at 540 nm. ** *p* = 0.02; (**C**) levels of proteins associated with epithelial–mesenchymal transition (EMT). Cells were seeded in low-attachment plates in triplicates. After 14 days, spheroids formed were disassembled and the levels of E-cadherin and vimentin assessed using flow cytometry. Percentage of E-cadherin and vimentin-positive cells are presented. The average of three experiments is shown. The *T*-test was used for statistical analysis. * *p* = 0.00, *** *p* = 0.03.

Wound healing assays were performed using mitomycin C treated parental cells. No significant differences were observed in cell cycle arrest induced by mitomycin C, neither between PHK18A1 and 18B1 (*p* = 0.65) nor between parental PHKs and PHK18A1 (*p* = 0.84) or parental PHKs and PHK18B1 (*p* = 0.50) (Supplementary Figure S1A). At 12 h following wounding, no differences in migration between PHK18A1 and PHK18B1 were observed (*p* = 0.28) (Figure 5A). However, we detected important differences regarding cell migrations patterns among HPV-18 variants (videos in Supplementary Videos S1–S6). While PHK18A1 migrated collectively, i.e., we observed a group of cells migrating together toward the wound area, PHK18B1 cells tended to move more individually. Regarding invasion potential, PHK18A1 (mean absorbance 0.99) had a significantly higher invasion potential than PHK18B1 (mean absorbance 0.81) (*p* = 0.00). Moreover, while radial and central invasion was observed for PHK18A1, PHK18B1 exhibited solely radial invasion (Figure 5B). The invasion of large clusters of PHK18A1 throughout inserts was also observed, whereas PHK18B1 invaded in small groups of cells.

### 3.8. PHK18A1 and PHK18B1 Spheroids Differ in Epithelial–Mesenchymal Transition (EMT) Phenotype

Epithelial to mesenchymal transition (EMT) involves ECM degradation and is characterized by loss of E-cadherin concomitant with augmented vimentin levels [32]. To evaluate whether the greater invasion ability of PHK18A1 was associated with EMT, the levels of both proteins were assessed among spheroids formed after plating in low-attachment plates. We observed that parental cells presented an epithelial phenotype (more cells expressing E-cadherin), while immortalized PHKs harbored a mesenchymal phenotype as inferred by the higher number of cells expressing vimentin. Significant higher levels of cells expressing vimentin were observed among PHK18A1 (42.8%), in comparison to PHK18B1 (22.3%) (*p* < 0.00) (Figure 5C, Supplementary Figure S2).

### 3.9. PHK18A1 and PHK18B1 Form Similar Epithelia in Raft Cultures

**Figure 6:**
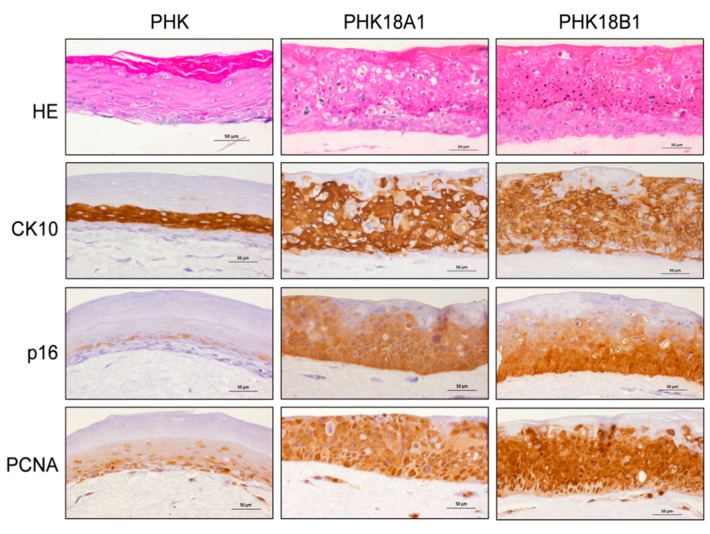
Organotypic cultures obtained from immortalized PHK18A1 and PHK18B1. Shown are representative micrographs of HE stained tissues, in addition to IHQ to assess the levels of CK10, p16, and PCNA. Figures derived from a representative of two independent assays performed.

PHK18A1 and PHK18B1 were used to establish organotypic raft cultures that allow for differentiation similar to that observed in epithelial tissues [33]. Ordered stratification reminiscent of the normal skin was observed in raft cultures derived from parental PHKs, where we observed basophilic undifferentiated basal cells, in addition to several layers of eosinophilic suprabasal cells (Figure 6). On the other hand, tissues obtained from PHK18A1 and PHK18B1 were hyperplastic and presented abnormal stratification preventing the discrimination between basal and suprabasal layers. No differences were observed among cultures derived from PHK18A1 and PHK18B1 concerning epithelial thickness or morphology.

CK10 levels were uniformly detected throughout cells of all suprabasal epithelium layers of tissues derived from parental PHKs or from immortalized PHKs, regardless of the HPV-18 variant. CK10 is a marker of differentiation, as well as of hyperplasia reserve cells, squamous metaplasia, and the cervical transformation zone [34]. No differences were observed in p16 or PCNA staining patterns between PHK18A1 and PHK18B1 (Figure 6). It is noteworthy that in tissues derived from PHK18A1 and PHK18B1 high levels of CK10 and PCNA were concomitantly detected in most cells within suprabasal layers, indicating that E6 and E7 proteins of the two HPV-18 variants were capable of inducing both proliferation and differentiation in the same cell. It is noteworthy, however, that PCNA levels seem to be higher in PHK18B1 rafts, in comparison to PHK18A1 derived tissue. Taken together, our data show no significant differences regarding morphology and levels of the different proteins analyzed between PHK18A1 and PHK18B1-derived epithelia that could indicate lesions with different tumorigenic potential.

## 4. Discussion

We investigated E6/E7 functional characteristics relevant to carcinogenesis of two HPV-18 sublineage variants commonly detected in our population [6] in the genetic background of natural host cells using an in vitro model that resembles persistent infection. PHK18A1 reached *p*30 at a significantly earlier time point, compared to PHK18B1, and an evident crisis prior to immortalization was not observed. Our data are in line with those of Lace et al. [35], who also did not observe a clear crisis pre-immortalization period when PHKs of different anatomical origins were infected with HPV-16 or HPV-18 whole genome but contrasts to previous results from our group and others regarding PHKs immortalized by E6 and E7 of different HPV-16 variants in which a clear “crisis” was observed between *p*10–15 [7,8].

During HPV-induced carcinogenesis, HR-HPV E7 degrades pRb, releasing the E2F transcription factor that induces cell proliferation [18]. Furthermore, HR-HPV E6 induces the degradation of p53 and activates hTERT [36], which are crucial for infected cells to abrogate growth arrest and apoptosis induced by DNA damage, and to extend the lifespan of PHKs, respectively. E6 protein levels fluctuated during passages in culture in both PHK18A1 and PHK18B1, and p53 levels were inversely correlated to those of E6. As HPV-18 E6*I has been shown to inhibit E6-mediated degradation of p53, we hypothesize that in our model, although E6*I mRNA levels did not vary between variants, at *p*30 in both PHK18A1 and PHK18B1, E6*I protein levels are higher than full-length E6, which, however, can direct degradation of other cellular proteins in the absence of full-length E6 protein to sustain immortalization [37]. Actually, although p53 and pRB are the most studied interactors of E6 and E7, respectively, many other cellular targets of both viral proteins reported [38] can respond to the differences observed between PHK18A1 and PHK18B1 throughout this study. Unfortunately, under our experimental conditions, we were unable to detect E6*I protein in any of the samples, limiting conclusions. Our data contrast with a previous report in which high levels of E6*I transcripts were detected solely in MCF-7 and C33 cells transfected with E6 of an HPV-18 B1 sublineage variant [23,24]. Discrepancies observed between studies most probably rely upon differences regarding the models and the genetic background of the cells used, hindering any further consideration.

The induction of growth arrest is an important cellular response to DNA damage which avoids accumulation of mutation driving carcinogenesis [39]. Both HPV-18 variants were able to overcome AD-induced growth arrest, once the rate of cell in the proliferative S phase increased after treatment. AD has also been shown to be a potent inducer of apoptosis [40]. The higher levels of active caspases 3 and 7 and sub-2N cells observed in AD treated PHK18B1 indicate that PHK18A1 were more resistant to AD-induced apoptosis. This property may confer to HPV-18 A1 sublineage variant a higher oncogenic potential once reduced susceptibility to apoptosis supports tumor growth [41].

The ability of single cells in forming colonies through clonal expansion is a largely used measure of oncogenic potential [42]. Some approaches were used here and overall show that PHK18A1 were able to form a higher number and larger colonies and spheroids in monolayer and 3D cultures, respectively. Of importance, our study highlights qualitative differences among PHKs immortalized by the different variants: in contrast to PHK18A1, which formed more compact colonies and spheroids of firmly grouped cells, PHK18B1 tended to form structures of noncohesive cellular clusters, which resembles colonies and spheroids formed by parental PHKs. We hypothesize that interactions of E6/E7 of the different HPV-18 variants with different cellular proteins, for instance, PTEN [25], may explain the differences observed. Although in a different genetic background, it was also previously observed a higher number of colonies arising from NIH3T3 cells expressing solely E6 of the HPV-18 A1 variant grown in soft agar, compared to other variants [23]. Although migration was similar among variants, migration patterns were also different: PHK18A1 tended to migrate in clusters of cells as commonly observed in epithelial tumors, whereas PHK18B1 migrated individually similar to solid stromal tumors. In fact, a broad spectrum of migration and invasion mechanisms have been observed among cancer cells [32]. It was hypothesized that the advantage of migrating in clusters over migrating individually confers protection of inner cells from immunological attack, thereby increasing the efficiency of tumor invasion and survival. Nevertheless, further studies are needed to identify the mechanisms underlying each type of cell migration observed here. Our data contrast to that of Fragoso-Ontiveros et al. [43], who observed significantly higher migration potential of cells expressing only E6 the HPV-18 B1 variant, compared to the A1 counterpart. However, direct comparisons are also hindered by differences in cell assay models.

PHK18A1 showed an enhanced capacity for invasion through a collagen matrix when compared to PHK18B1. We presume that the interaction of viral and target proteins that compose the basement membrane, interstitial stroma, and/or extracellular matrix may contribute to invasion during HPV-induced carcinogenesis. For instance, it was shown that the HPV-16 E6 PDZ binding motif significantly contributes to Caski and HeLa cells migration and invasion [44]. We suggest that the higher and lower number of cells expressing vimentin and E-cadherin, respectively, observed in PHK18A1 may contribute, at least in part, to the higher invasion potential observed for these cells, once downregulated E-cadherin is associated with decreased cell–cell adhesion, facilitating EMT and invasion into adjacent tissues [32].

E6 expression is also crucial for PHKs to resist terminal differentiation induced by serum and calcium [45]. We observed that, when cells were maintained in a differentiation-inducing medium, PHK18B1 exhibited greater resistance to differentiation, compared to PHK18A1. However, we may not exclude the chance that these cells, by a mechanism not elucidated here, have a higher ability to survive when grown in a calcium-rich medium. Furthermore, once MTT, which was used to visualize colonies, is processed by the mitochondria, the possibility exists that E6/E7 of the HPV-18 variants analyzed differently impact cell respiration. Although some studies have evaluated the role of E6 and E6*I in mitochondrial activity associated with oxidative stress and cell death, there are no reports regarding the influence of mitochondrial functions on the differentiation of HPV-infected keratinocytes [23,46]. It should be noted also that resistance to differentiation and immortalization may develop by independent pathways.

Finally, we did not observe any evident differences between PHK18A1 and PHK18B1 in forming epithelia in organotypic culture: both showed hyperplasia, in addition to high levels of CK10, PCNA, and p16 throughout the tissue, compatible with cervical intraepithelial neoplasia [20,47]. Nevertheless, it is feasible that the different parameters evaluated were not sufficient to reveal lesions with different degrees of aggressiveness. Furthermore, although the expressions of HR-HPV E6 and E7 induce alterations in keratinocyte differentiation in organotypic cultures [47,48,49], this model may not be adequate to unravel differences attributable to these variants regarding association to different histological subtypes of CC. In fact, although PHKs have been extensively used in the last decades in functional studies aiming to understand the mechanisms of HPV-induced cervical carcinogenesis better, we believe that pathological differences between cervical SCC and ADC would be better observed in the context of squamocolumnar junction cells, which are believed to constitute the source of cervical cancer [50].

HPV-18 naturally occurring variants might exhibit other biological differences than those evaluated in this study, which may affect their pathogenic potential, including differences in the host immune response. However, as a whole, our data indicate that PHK18A1 exhibits a phenotype that more closely resembles transformed cells, in comparison to PHK18B1. Further studies are necessary to understand the underlying biochemical mechanisms and pathways behind the observed differences.

## 5. Conclusions

PHKs immortalized by E6/E7 of HPV-18 A1 and B1 variants were characterized. Regarding hallmarks of cancer cells, PHK18A1 showed higher oncogenic potential. Furthermore, we highlight qualitative differences among immortalized PHKs, which might impact their association to different CC histological subtypes.

## Data Availability

The data presented in this study are available on request from the corresponding author.

## Supplementary Materials

The following are available online at https://www.mdpi.com/article/10.3390/v13061114/s1, Table S1: Sequence of primers used for qRT-PCR, Table S2: Nucleotide and aminoacidic sequence variation among HPV-18 variant, Figure S1: Migration potential of parental PHKs and immortalized PHK18A1 and PHK18B1, Figure S2: EMT phenotype of immortalized PHK18A1 and PHK18B1., Videos S1–S6: Migration potential of parental PHKs and immortalized PHK18A1 and PHK18B1.
