# Supplementary material for: E6/E7 Functional Differences among Two Natural Human Papillomavirus 18 Variants in Human Keratinocytes
Source: Viruses. 2021 Jun 10;13(6):1114. doi: 10.3390/v13061114 (PMC8228617; doi:10.3390/v13061114)
Supplement: Supplementary file 1 [file viruses-13-01114-s001.zip › revised-Nunes et al.-supplementary tables.pdf]

Supplementary Table 1. Sequence of primers used qRT-PCR.

transcript	NCBI Reference or Genbank ID	nt position	Orientation	Sequence
S18	NM_014046	218 - 238	Forward	CCAGGAGCGATATGGTTCTCG
S18	NM_014046	365 - 343	Reverse	CAACTTGTGATCTCGACAGATGG
E7	X05015	750 - 768	Forward	CCGAACCACAACGTCACAC
E7	X05015	852 - 833	Reverse	TGCTGGAATGCTCGAAGGTC
E6 + E6*	X05015	111 - 130	Forward	CGCTTTGAGGATCCAACACG
E6 + E6*	X05015	180 - 160	Reverse	GCAGTGAAGTGTTCAGTTCCG
Only E6	X05015	506 - 525	Forward	TAGAGGCCAGTGCCATTCGT
Only E6	X05015	578 - 557	Reverse	TACTTGTGTTTCTCTGCGTCGT

Supplementary Table 2. Nucleotide and aminoacidic sequence variation among HPV-18 variants. E6 and E7 nucleotide and aminoacidic positions where variations between the A1 and B1 variants were detected are depicted. Cases of aminoacid changes are indicated in parenthesis. nt- nucleotide. aa – aminoacid. pos – position.

Gene/Protein	nt pos/aa pos	nt A1	aa A1	nt B1	aa B1
	251/49	T	Phe	C	Phe
	266/54	G	Val	A	Val
	317/71	T	Phe	C	Phe
	374/90	G	Leu	A	Leu
**E6**	482/126	A	Arg	G	Arg
	485/127	T	Phe	C	Phe
	491/129	C	Asn	A	Lys
	548/148	A	Glu	G	Glu
	549/149	C	Arg	A	Arg
	593/2	C	His	T	Tyr
**E7**	640/17	C	Pro	T	Pro
	751/54	C	Ala	T	Ala
