# Supplementary figures and images for: E6/E7 Functional Differences among Two Natural Human Papillomavirus 18 Variants in Human Keratinocytes
Source: Viruses. 2021 Jun 10;13(6):1114. doi: 10.3390/v13061114 (PMC8228617; doi:10.3390/v13061114)

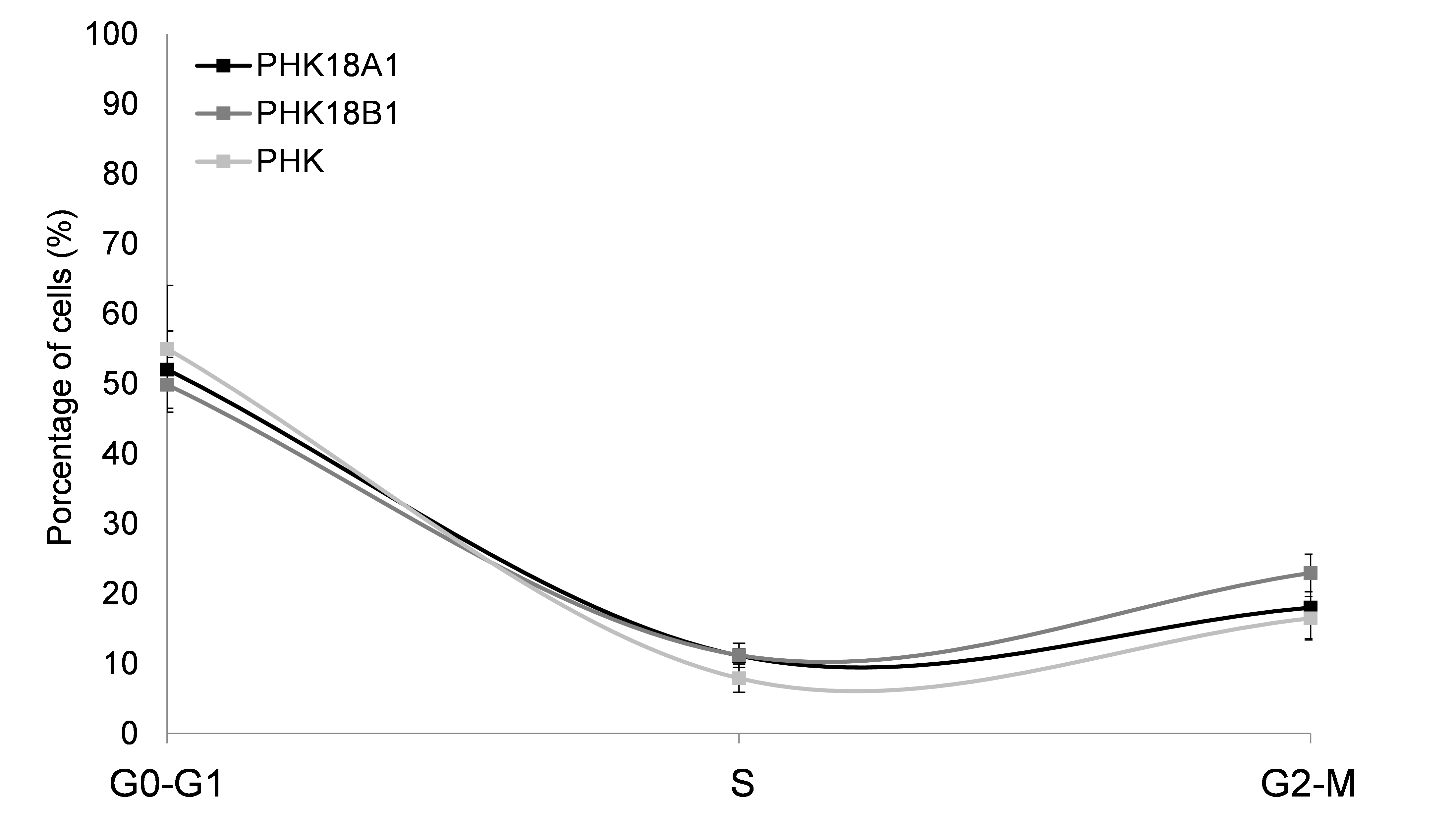

Supplement: Supplementary file 1 [file viruses-13-01114-s001.zip › FigureS1A_600dpi.tif]

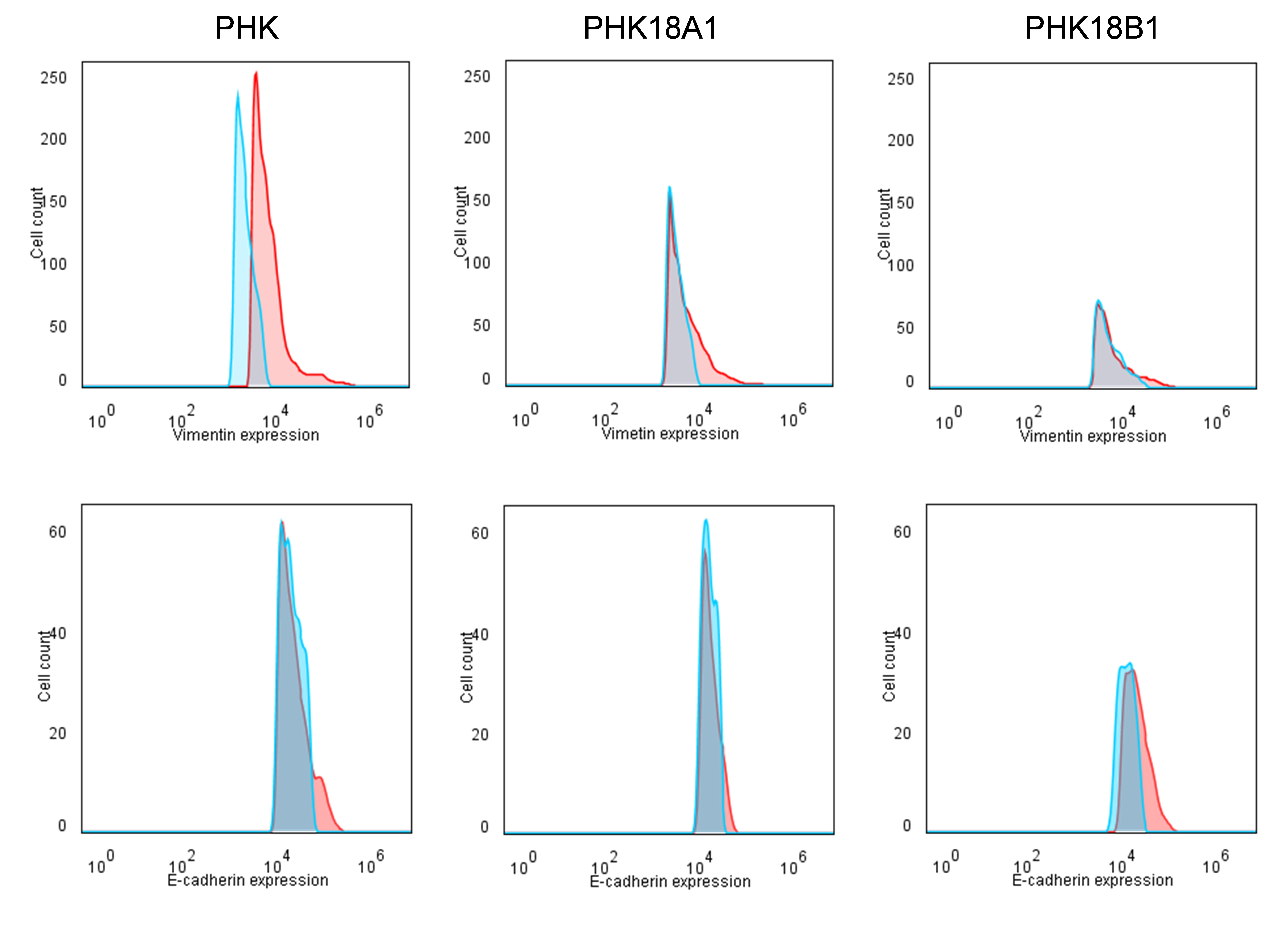

Supplement: Supplementary file 1 [file viruses-13-01114-s001.zip › FigureS2_600dpi.tif]
